# Relationship between chronotype and mental behavioural health among adolescents: a cross-sectional study based on the social ecological system

**DOI:** 10.1186/s12888-023-04879-6

**Published:** 2023-06-06

**Authors:** Yi Zhang, Zhengge Jin, Shuqin Li, Huiqiong Xu, Yuhui Wan, Fangbiao Tao

**Affiliations:** 1 Department of Maternal, Child and Adolescent Health, School of Public Health, Anhui Medical University, No 81 Meishan Road, Hefei, 230032 Anhui China; 2 NHC Key Laboratory of Study On Abnormal Gametes and Reproductive Tract, No 81 Meishan Road, Hefei, 230032 Anhui China; 3 MOE Key Laboratory of Population Health Across Life Cycle, No 81 Meishan Road, Hefei, 230032 Anhui China

**Keywords:** Chronotype, Mental health, Health risk behavior, Social ecological risk factor, Adolescents

## Abstract

**Background:**

Health risk behaviors (HRBs) is a kind of phenomenon behavior that often occurs in adolescence, and also often appears in clusters. Previous studies suggested an association between social ecological risk factors (SERFs) and HRBs. This study explored 1) whether chronotype moderates the risk of HRBs associated with SERFs and 2) whether mental health is a mediator in this relationship.

**Methods:**

Adolescents were recruited from 39 junior or senior schools (three cities, 13 schools per city) using a multistage cluster sampling method conducted between October, 2020 and June, 2021. The Social Ecological System, Morningness–Eveningness Questionnaire, Brief Instrument on Psychological Health Youths, and Youth Risk Behavior Surveillance questionnaires were used to measure the SERFs, chronotype, mental health and HRBs. Latent category analysis was used to explore the clustering mode of HRBs. The primary exposure was SERFs, and the primary outcome was HRBs; chronotype was a moderator, and mental health was a mediator. The multivariable logistic regression model was used to determine the relationship between SERFs and chronotype and mental behavioral health status. Mediation moderate analysis using the PROCESS method was used to explore the relationship between these variables. Sensitivity analysis was conducted to evaluate the robustness of the model.

**Results:**

In total, 17,800 individuals were initially enrolled. After excluding 947 individuals with invalid questionnaires, 16,853 participants were finally included in the analysis. The mean age of participants was 15.33 ± 1.08 years. After adjusting for covariates, multivariable logistic regression found that high levels of SERFs (odds ratio [OR] = 10.10, 95% confidence interval [CI]: 8.88–11.43, *P* < 0.01), intermediate chronotype (OR = 5.24, 95% CI: 4.57–6.01, *P* < 0.01), and eveningness (OR = 1.83, 95% CI: 1.64–2.05, *P* < 0.01) were associated with higher HRBs frequency. This study also assessed the interaction between chronotype, SERFs and HRBs (OR = 27.84, 95% CI: 22.03–35.19, *P* < 0.01) and mental health (OR = 18.46, 95% CI: 13.16–25.88, *P* < 0.01). The moderated mediation analyses examined the relationship between chronotype, SERFs, mental health and HRBs.

**Conclusions:**

SERFs may be important variables in measuring the effect of the adolescent psychosocial environment on HRBs; this effect is mediated by mental health and moderated by chronotype.

**Supplementary Information:**

The online version contains supplementary material available at 10.1186/s12888-023-04879-6.

## Background

Health risk behaviors (HRBs) among adolescents are the subject of global interest. Moreover, mental and behavioral health outcomes, including depression and disorders related to the use of alcohol and other substances, have gained much attention in recent years [1]. Adolescent HRBs generally cause direct or indirect damage to the health, well-being, and quality of life of adolescents [2]. A cohort study identified two latent classes of HRBs: a risky-behavior class (men: 20.0% and women: 23.6%), including smoking and binge drinking, and a less-risky-behavior class [3]. Other studies have reported that approximately 50–60% of adolescents engaged in at least two HRBs [4, 5]. In a previous study, involving 22,628 middle-school students in six cities in China, our research group found that approximately 36% of the adolescents had moderate- to high-risk HRBs [4]. Taken together, these evidence shows that adolescent HRB cooccurrence and clustering are common [4, 6, 7].

Evidence indicates that behaviors and lifestyles formed during adolescent stage maintain a "trajectory" into adulthood and affect lifelong health, potentially increasing the risk of morbidity and mortality [3, 8, 9]. Although public health researchers claim that adolescent HRBs stem from factors at the individual, family, or school level, tthe persistence of high rates of HRBs in modern societies seems to be explained by more than these factors alone. Therefore, it is important to identify factors that contribute to HRBs based on a theory that can account for multiple HRBs.

Some studies have suggested that cumulative risk models can be used to understand how psychosocial environmental factors affect long-term outcomes. Among these, social ecological system (SES) models in public health [10] and their extensions, such as the theory of ecological development [11, 12], cumulative ecological-transactional risk perspective [13], and socioecological psychology [14], suggest the existence of multiple social ecological risk factors (SERFs) that influence HRBs in adolescents. Therefore, these theoretical approaches support the occurrence of adolescent HRBs. Recent research has found that risk and protective factors at different levels of influence (such as family, school, and peers) are directly or indirectly associated with multiple mental, emotional, and behavioral issues [11, 15, 16].

Chronotype, or morning vs. evening preference, is an intrinsic biological trait defined by sleep–wake cycles and changes in morning and evening attention levels, which is also correlated with HRBs in adolescents [17]. The term refers to the individual’s time preference in terms of sleep vs. activities and is generally divided into "morningness," "intermediate chronotype," and "eveningness". Morningness chronotypes tend to have earlier sleep schedules and prefer to be active in the morning, whereas eveningness chronotypes tend to sleep late and function best in the late afternoon or evening [18]. Tavernier et al. analyzed the association between eveningness and substance use in a longitudinal study of 1^st^-year students and found that substance use was more frequent among "nocturnal" college students during follow-up [19]. Previous studies have suggested that morningness is a protective factor against negative mood [20, 21].

Furthermore, studies on the interaction of environmental, socio-ecological, and behavioral factors with the human circadian cycle under ecological conditions have undergone some preliminary validation. The diathesis stress model, combined with a SES framework, claims to address the HRBs ecology-diathesis stress model in understanding HRBs, which we consider as an integrated society, one that recognizes the complex and dynamic natures of HRB involvement across multiple settings (e.g., individuals, families, communities, schools, and culture) over time. We hypothesized that there is an association between SERFs, chronotypes, and adolescent HRBs and mental health. Although methods of SERF assessment are gradually developing, most studies have focused on a single HRB [22] or on HRB cooccurrence and clustering [23]. There are limited comprehensive reports on current research into policy, culture, and chronosystems of spatial dimensions. Moreover, to date, the relationship between chronotype, HRBs, and mental health among cumulative SERF environments has not been explored.

The above-mentioned studies provide some indirect evidence of the association between SERFs and HRBs, and a comprehensive model incorporating evidence on the association between SERFs and HRBs is required. Furthermore, the cooccurrence and clustering phenomena of adolescent HRBs should be thoroughly examined. We conducted a large, multicenter, cross-sectional study across different schools in three Chinese cities. This study explored the interaction between HRBs and SERFs and examined the role of cumulative SERFs to determine whether adolescent chronotypes combined with cumulative SERFs influence HRBs and mental health [24]. Specifically, the aims of this study were to establish the following: 1) whether chronotype moderates the risk of HRBs through SERFs; 2) whether mental health is a mediator of this relationship; 3) whether there is an interaction between chronotype and SERFs and HRBs and mental health; and 4) whether there is a mediating effect of mental health on the relationship between HRBs and SERFs, in addition to the moderating role that chronotype plays in the relationship.

## Methods

### Study design

This was a cross-sectional study using an extensive survey procedure to assess the sociodemographic and psychosocial environmental factors that contribute to health and HRBs of the general population [25, 26]. The study was designed and reported according to the Strengthening the Reporting of Observational Studies in Epidemiology checklist.

### Settings

We used data from a collaborative cross-province survey of adolescent health and well-being that examine health and well-being of adolescents in their social context every 2 years in three-to-four cites in China [25, 26]. Data were collected in all participating cities through self-reported school-based surveys using a standard methodology described in detail in the study protocol [25, 26]. A cross-sectional study design was used to conduct nationwide sample surveys across China. The participants were high-school and junior high-school students in China, who were surveyed between October, 2020 and June, 2021 using multistage stratified cluster random sampling. The design and data collection procedures were approved by the Ethics Committee of the Anhui Medical University (reference no: 20200965). Written informed consent was obtained from the parents or guardians of all students. All methods used in this study were in accordance with the relevant guidelines and regulations. Detailed methods are shown in the Supplementary Methods (page 1).

### Inclusion criteria

The inclusion criteria were as follows: 1) informed consent of the participant and his or her guardian was obtained; 2) the participant was a junior high or hig- school student (aged 12–20 years); 3) the participant had no history of mental illness; and 4) the participant was a student attending a chosen school.

### Exclusion criteria

**Fig. 1:**
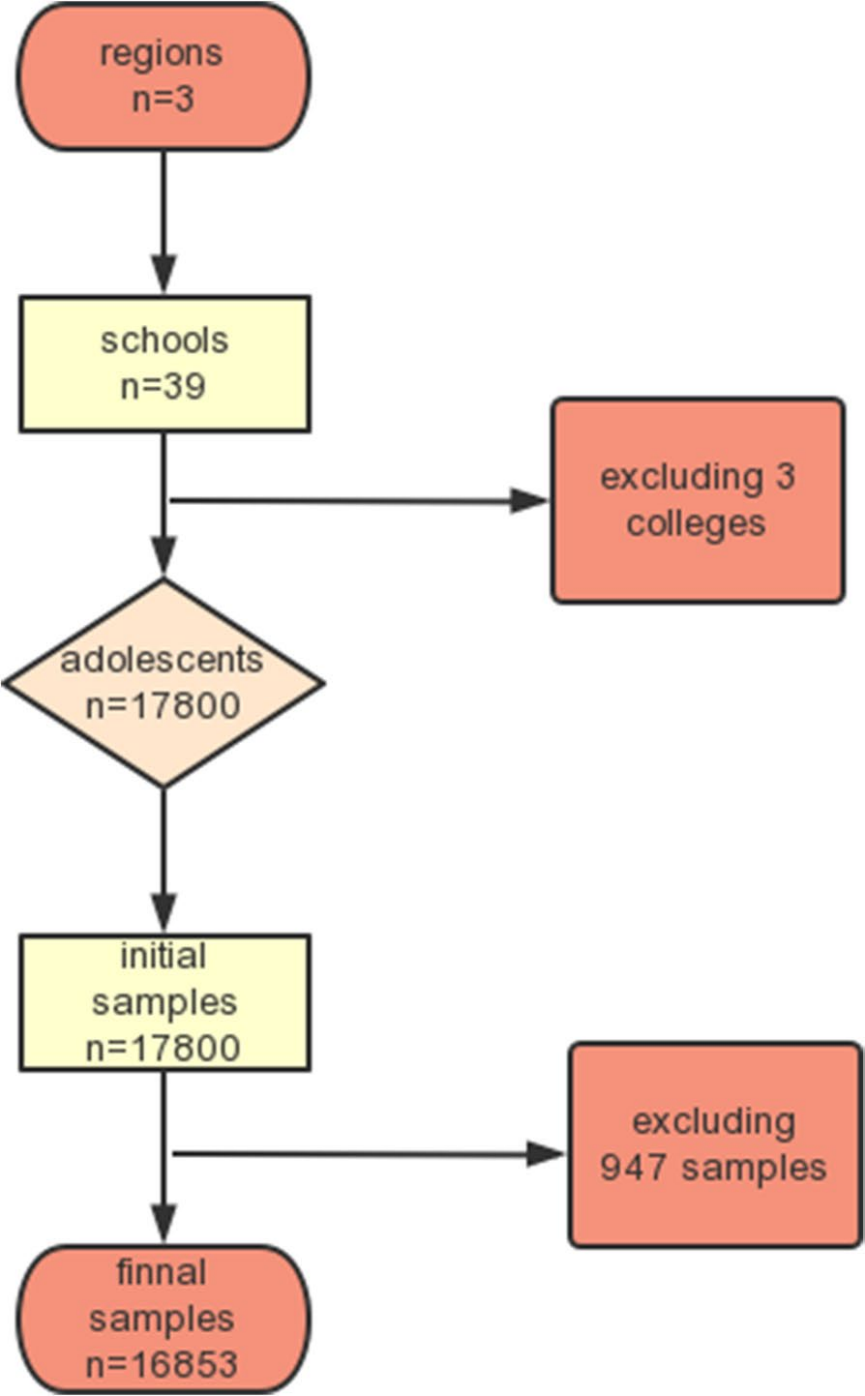
Flow chart of participant recruitment

The exclusion criteria were as follows: 1) failure to obtain informed consent for participation in the study; 2) participant was a college student; 3) failure to submit the questionnaire; and 4) the participant had a congenital or acquired immunodeficiency. The flow chart of participant recruitment in this study is shown in Fig. 1.

### Exposure

#### Chronotype

Chronotype was monitored using the shortened version of the Morningness–Eveningness Questionnaire (MEQ). Morning or evening preference was categorized as morningness (“definitely a morning person”), intermediate chronotype (“between a morning person and an evening person”), and eveningness (“definitely an evening person”) [27]. The groups were defined using scoring as follows: 4–7, definite eveningness; 8–11, medium eveningness; 12–17, intermediate chronotype; 18–21, medium morningness; and 22–25, definite morningness. The Cronbach’s α coefficient for the MEQ was 0.628 in this study.

#### Social ecological risk factors

The SERFs consisted of 50 items, which were divided into seven dimensions, including individual, family, school, community, policy, culture, and chronosystem profiles. Detailed methods regarding the SERFs are shown in Supplement Table 1 (pages 4–5).

### Outcomes

#### Health risk behaviors

HRBs were measured using the Youth Risk Behavior Surveillance system [28], according to methods described in our previous study [26]. We extracted 15 HRB types: smoking, alcohol consumption, skipping breakfast, excess weekday and weekend screen time, physical inactivity, fast food consumption, takeout consumption, sugar sweetened beverage (SSB) consumption, dietary scarcity of vegetables and fruits, suicidal thoughts, suicidal plans, suicide attempts, and non-suicidal self-injury (NSSI). Detailed methods are shown in the Supplementary Methods (pages 1–4).

#### Mental health

The Brief Instrument on Psychological Health of Youths (BIOPHY) scores the above-mentioned 15 HRB items on a 6-point scale. Response scores range from 1 = “It lasts for more than 3 months”; 2 = “It lasts for more than 2 months”; 3 = “It lasts for more than 1 month”; 4 = “It lasts for more than 2 weeks”; 5 = “It lasts for more than 1 week”; and 6 = “It lasts less than a week or not.” The analysis of the total BIOPHY score was divided into high- and low-level categories, according to the 90^th^ percentile of the total score [29]. The Cronbach’s α coefficient for the BIOPHY was 0.958 in this study.

### Covariates

Age, sex, parental education level, residential area, whether the participant was an only child, family economic status, number of friends, and academic record were included as covariates.

### Statistics analysis

Results for categorical variables were described using frequency and percentage and analyzed using T-test (dichotomous) and Chi-square test (multivariate). Continuous variables were described using mean and standard deviation and analyzed using one-way analysis of variance. In order to identify clusters of HRBs, latent class analysis (LCA) was used to identify homogeneous, mutually exclusive "patterns" of 15 HRBs using Mplus 7.4, according to the method described in a previous study [25]. Multivariable logistic regression was used to examin the relationships between SERFs, chronotype and mental health, HRBs and presented as adjusted odds ratios (aORs) with 95% confidence intervals (CIs). Mediation moderate analysis was used according to the PROCESS method to explore the relationship between SERFs, chronotype, mental health and HRBs. All analyses were conducted using the Statistical Package for the Social Sciences (SPSS) software, version 23.0. After preliminary data sorting, missing data was processed in SPSS 23.0. In general, the data missing rate of this study was very low, and the missing rate of each item was less than 1%. Therefore, the multiple imputation method was adopted in SPSS 23.0 to process missing data at the project level. Our sensitivity analysis method is shown in the Supplement Methods.

## Results

### Class enumeration

Supplement Table 2 (page 7) depicts the fit statistics for the one- to nine-class models. The three-class solution was chosen as the final, best-fitting model based on its lower AIC (akaike information criterion), as well as lowest BIC (bayesian information criterion) and aBIC (bayesian information criterion) values. Moreover, we listed the trend of aBIC values and found that the three-class solution had the most significant decrease in aBIC values. With regard to model 3, bootstrap validation procedures also demonstrated a good fit (*p* < 0.001). All remaining results were reported specifically to the three-class solution.

### Characteristics of the final four-class model

**Fig. 2:**
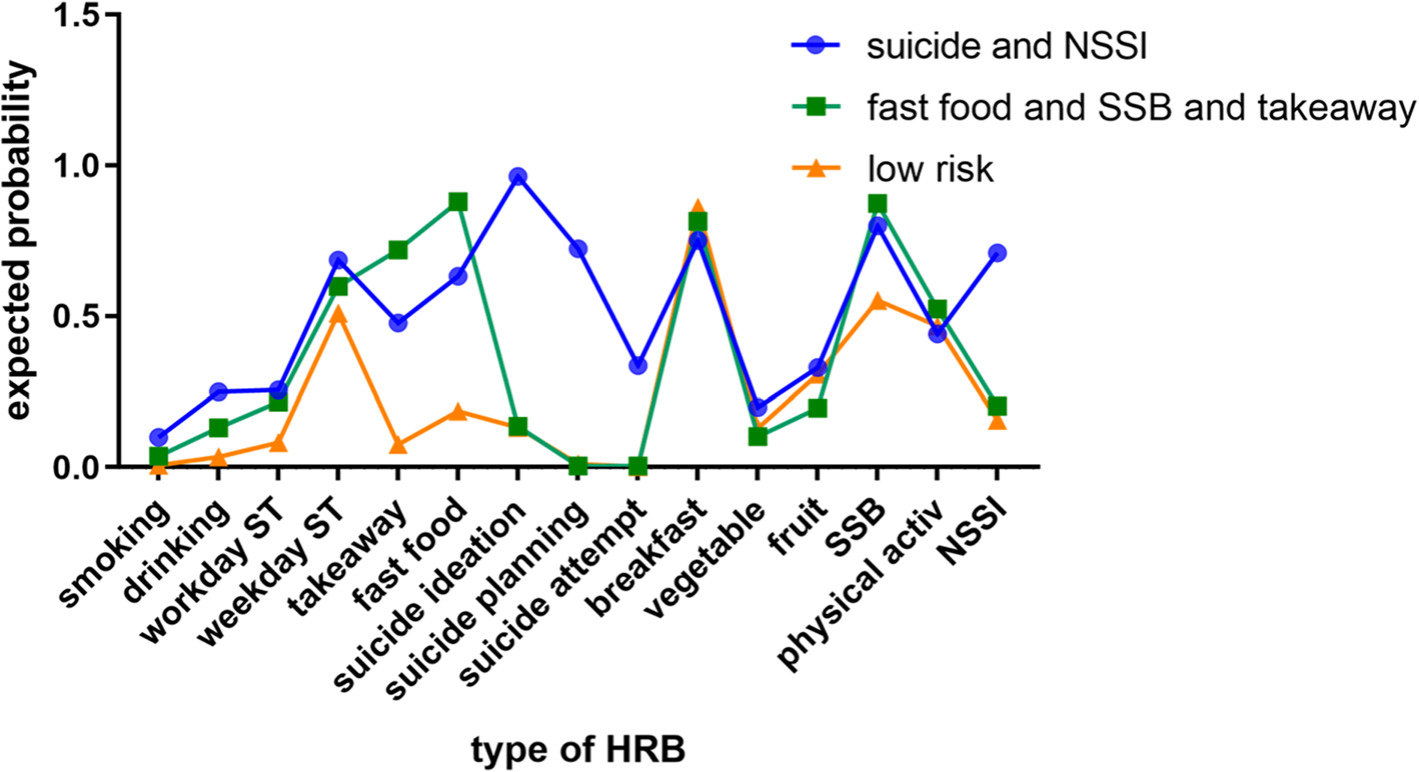
Classes of health risk behaviors

Figure 2 shows the three-class model of HRBs and item-response probabilities for the 15 HRBs for each class. Class 1 was characterized by a high probability of exposure to each of the 15 HRBs; therefore, we labeled this latent class “suicide and NSSI” (21.3%). Class 2 comprised individuals with medium-level HRBs and was labeled as “fast food, SSB, and takeout (dietary behaviors)” (27.9%). Class 3 was characterized by a low probability of exposure to each of the 15 HRBs and was labeled as “low risk” (50.8%).

### Prevalence of demographic variables, chronotypes, and SERFs on HRBs and mental health

In total, 17,800 junior and senior middle school students aged 10 to 20 years were recruited in this study using a health survey of adolescents in junior and senior middle schools (grades 7–12). We checked the quality of all questionnaires and excluded those with regular answers, contradictory answers (obvious logical errors), or more than five consecutive unanswered questions. After excluding 947 (5.32%) questionnaires, 16,853 questionnaires were included in the analysis, and the efficiency rate was 94.68%. The mean participant age was 15.33 ± 1.08 years.

Sample characteristics for covariates, SERFs, and chronotypes are described in Table 1 for each of the three HRB groups. Our results showed that higher total SERF and eveningness were associated with higher suicide and NSSI scores (total SERF: *χ*^2^ = 1,737.84 and eveningness: *χ*^2^ = 751.52). Girls were more likely to have higher suicide and NSSI scores (*χ*^2^ = 86.54) than boys. Lower parental education levels were also found to be correlated with higher suicide and NSSI scores (father: *χ*^2^ = 201.65 and mother: *χ*^2^ = 203.95).

**Table 1:** The prevalence characteristics and social ecological risk factor and chronotype of HRBs

	Total	Suicide and NSSI	Dietary behaviors	Others	*χ*^2^ value
Age	14.99 ± 1.64	15.23 ± 1.72	15.29 ± 1.77	14.99 ± 1.64	38.41^**^
Gender					86.54^**^
Male	8390(49.8)	1551(18.5)	2463(29.4)	4376(52.2)	
Female	8463(50.2)	2041(24.1)	2173(25.7)	4249(50.2)	
Residential area					48.19^**^
Country	2593(15.4)	534(20.6)	588(22.7)	1471(56.7)	
Town	3212(19.1)	681(21.2)	879(27.4)	1652(51.4)	
Urban	11,048(65.6)	2377(21.5)	3169(28.7)	5502(49.8)	
Only child					128.68^**^
Yes	5710(33.9)	1180(20.7)	1876(32.9)	2654(46.5)	
No	11,143(66.1)	2412(21.6)	2760(24.8)	5971(53.6)	
Father's education					201.65^**^
No father	189(1.1)	94(49.7)	30(15.9)	65(34.4)	
Below primary school level	505(3.0)	148(29.3)	90(17.8)	267(52.9)	
Primary school	1325(7.9)	326(24.6)	298(22.5)	701(52.9)	
Junior high school	6022(35.7)	1245(20.7)	1542(25.6)	3235(53.7)	
Senior high school	4902(29.1)	1010(20.6)	1444(29.5)	2448(49.9)	
College school	3910(23.2)	769(19.7)	1232(31.5)	1909(48.8)	
Mother's education					203.95^**^
No mother	115(0.7)	52(45.2)	22(19.1)	41(35.7)	
Below primary school level	744(4.4)	195(26.2)	137(18.4)	412(55.4)	
Primary school	1679(4.4)	368(21.9)	356(21.2)	955(56.9)	
Junior high school	6036(35.8)	1256(20.8)	1522(25.2)	3258(54.0)	
Senior high school	4634(27.5)	1004(21.7)	1397(30.1)	2233(48.2)	
College school	3645(21.6)	717(19.7)	1202(33.0)	1726(47.4)	
Family economic status					295.65^**^
Very bad	475(2.8)	190(40.0)	85(17.9)	200(42.1)	
Worse	1644(9.8)	475(28.9)	85(18.0)	873(53.1)	
Medium	11,677(69.3)	2307(19.8)	3206(27.5)	6164(52.8)	
Better	2431(14.4)	479(19.7)	844(34.7)	1108(45.6)	
Very good	626(3.7)	141(22.5)	205(32.7)	280(44.7)	
Friends number					350.98^**^
No	553(3.3)	249(45.0)	83(15.0)	221(40.0)	
1–2	4096(24.3)	1088(26.6)	940(22.9)	2068(50.5)	
3–5	6949(41.2)	1296(18.7)	1985(28.6)	3668(52.8)	
6 or more	5255(31.2)	959(18.2)	1628(31.0)	2668(50.8)	
Academic record					241.28^**^
Bad	4087(24.3)	1223(29.9)	1022(25.0)	1842(45.1)	
Medium	10,316(61.2)	1930(18.7)	2936(28.5)	5450(52.8)	
Good	2450(14.5)	439(17.9)	678(27.7)	1333(54.4)	
Individual					1258.24^**^
high	4883(29.0)	1847(37.8)	1112(23.6)	1924(39.4)	
medium	5610(33.3)	1065(19.0)	1638(29.2)	2907(51.8)	
low	6360(37.7)	671(10.6)	1960(30.8)	3729(58.6)	
Family					1452.72^**^
high	4644(27.6)	1821(39.2)	1042(22.4)	1781(38.4)	
medium	6380(37.9)	1250(19.6)	1861(29.2)	3269(51.2)	
low	5829(34.6)	521(8.9)	1733(29.7)	3575(61.3)	
School					909.37^**^
high	5251(51.2)	1807(34.4)	1296(24.7)	2148(24.9)	
medium	5732(34.0)	1119(19.5)	1554(27.1)	3059(53.4)	
low	5870(34.8)	666(11.3)	1786(30.4)	3418(58.2)	
Community					288.65^**^
high	4520(26.8)	1319(29.2)	1203(26.6)	1998(44.2)	
medium	6335(37.6)	1314(20.7)	1693(26.7)	3328(52.5)	
low	5998(35.6)	950(15.8)	1814(30.2)	3234(53.9)	
Public policy					196.64^**^
high	4597(27.3)	1283(27.9)	1238(26.9)	2076(45.2)	
medium	6320(37.5)	1288(20.4)	1713(27.1)	3319(52.5)	
low	5936(35.2)	1012(17.0)	1759(29.6)	3165(53.3)	
Culture					1025.14^**^
high	4860(28.8)	1660(34.2)	1473(30.3)	1727(35.5)	
medium	6186(36.7)	1206(19.5)	1792(29.0)	3188(51.5)	
low	5807(34.5)	717(12.3)	1445(24.9)	3645(62.8)	
Chronosystem					392.17^**^
high	2978(17.7)	1001(33.6)	763(25.6%)	1214(40.8)	
medium	5209(30.9)	1140(21.9)	1438(27.6%)	2631(50.5)	
low	8666(51.4)	1442(16.6)	2509(29.0%)	4715(54.4)	
Total score					1737.84^**^
high	5363(31.8)	2075(38.9)	1290(24.4)	1998(36.7)	
medium	5818(34.5)	1061(17.8)	1683(29.8)	3074(52.4)	
low	5672(33.7)	456(7.5)	1663(29.4)	3553(63.1)	
Chronotype					751.52^**^
Eveningness	2767(16.4)	1008(36.4)	876(31.7)	883(31.9)	
Intermediate chronotype	10,482(62.2)	2077(19.8)	3001(28.6)	5404(51.6)	
Morningness	3604(21.4)	498(13.8)	833(23.1)	2273(63.1)	

^*^p < 0.05, ^**^p < 0.01

Sample characteristics for covariates (sex, age, parental education level, family residence, only-child status, family economic status, number of friends, and academic performance), SERFs, and chronotypes are described in Supplement Table 3 (page 7) for each of the two mental health groups. Higher SERFs and eveningness were associated with higher negative mental health (total SERF: *χ*^2^ = 275.79 and eveningness: *χ*^2^ = 365.29). Boys were more likely to have mental health issues (*χ*^2^ = 26.41) than girls.

### Effect of SERFs and chronotypes on HRBs and mental health

As shown in Table 2, a high SERF level, intermediate chronotype, and eveningness were associated with higher suicide and NSSI scores (total SERF OR = 8.80, 95% CI: 7.81, 9.90; eveningness OR = 5.21, 95% CI: 4.56, 5.95; and intermediate chronotype OR = 1.75, 95% CI: 1.57, 1.96). After adjusting for covariates, these results were identical (all *p*-values < 0.01).

**Table 2:** The Logistic regression analysis between social ecological risk factors and clustering of health risk behaviors in adolescents

Social ecological risk factor	Model 1		Model 2		Model 3	
	Dietary behaviors	Suicide and NSSI	Dietary behaviors	Suicide and NSSI	Dietary behaviors	Suicide and NSSI
Individual						
High	1.10 (1.01,1.22) ^*^	5.34 (4.81,5.92) ^**^	1.21 (1.02,1.23) ^*^	5.35 (4.82,5.95) ^**^	1.29 (1.17,1.43) ^**^	4.87 (4.36,5.35) ^**^
Medium	1.08 (0.99,1.17)	2.04 (1.83,2.27) ^**^	1.08 (1.0,1.18) ^*^	2.06 (1.85,2.30) ^**^	1.18 (1.09,1.29) ^*^	1.98 (1.77,2.21) ^**^
Family						
High	1.21 (1.10,1.33) ^**^	7.02 (6.27,7.85) ^**^	1.20 (1.09,1.32) ^**^	7.21 (6.43,8.08) ^**^	1.36 (1.23,1.50) ^**^	6.57 (5.85,7.38) ^**^
Medium	1.17 (1.08,1.27) ^*^	2.62 (2.34,2.94) ^**^	1.18 (1.08,1.27) ^**^	2.76 (2.47,3.10) ^**^	1.28 (1.18,1.39) ^**^	2.60 (2.32,2.92) ^**^
School						
High	1.16 (1.06,1.26) ^*^	4.32 (3.89,4.79) ^**^	1.17 (1.07,1.28) ^**^	5.02 (4.51,5.59) ^**^	1.38 (1.26,1.52) ^**^	4.56 (4.08,5.10) ^**^
Medium	0.97 (0.89,1.06)	1.88 (1.69,2.09) ^**^	0.98 (0.90,1.06)	2.08 (1.86,2.32) ^**^	1.08 (0.99,1.18)	1.97 (1.76,2.20) ^**^
Community						
High	1.07 (0.97,1.17)	2.23 (2.01,2.46) ^**^	1.08 (0.98,1.18)	2.61 (2.35,2.89) ^**^	1.22 (1.11,1.35) ^**^	2.49 (2.24,2.77) ^**^
Medium	0.91 (0.84,0.98) ^*^	1.34 (1.22,1.48) ^**^	0.92 (0.84,0.995) ^*^	1.45 (1.32,1.60) ^**^	1.001 (0.92,1.09)	1.42 (1.28,1.57) ^**^
Public policy						
High	1.10 (1.003,1.21) ^*^	1.94 (1.76,2.14) ^**^	1.09 (0.99,1.20)	2.24 (2.02,2.49) ^**^	1.26 (1.15,1.39) ^**^	2.07 (1.86,2.30) ^**^
Medium	0.93 (0.86,1.01)	1.21 (1.10,1.33) ^**^	0.94 (0.87,1.03)	1.31 (1.19,1.45) ^**^	1.03 (0.94,1.12)	1.26 (1.14,1.39) ^**^
Culture						
High	2.13 (1.94,2.38) ^**^	4.84 (4.36,5.38) ^**^	2.41 (2.19,2.66) ^**^	5.88 (5.27,6.57) ^**^	2.25 (2.04,2.48) ^**^	5.73 (5.12,6.41) ^**^
Medium	1.43 (1.31,1.55) ^**^	1.92 (1.73,2.13) ^**^	1.54 (1.41,1.67) ^**^	2.21 (1.98,2.45) ^**^	1.51 (1.39,1.65) ^**^	2.17 (1.95,2.42) ^**^
Chronosystem						
High	1.17 (1.05,1.29) ^**^	2.65 (2.39,2.93) ^**^	1.18 (1.07,1.31) ^**^	2.70 (2.43,2.99) ^**^	1.21 (1.09,1.35) ^**^	2.53 (2.28,2.81) ^**^
Medium	1.01 (0.93,1.10)	1.40 (1.28,1.53) ^**^	1.03 (0.95,1.11)	1.40 (1.28,1.54) ^**^	1.05 (0.96,1.14)	1.36 (1.24,1.50) ^**^
Total score						
High	1.43 (1.31,1.57) ^**^	8.80 (7.81,9.90) ^**^	1.48 (1.35,1.62) ^**^	10.62 (9.39,12.0) ^**^	1.71 (1.55,1.89) ^**^	10.1 (8.88,11.43) ^**^
Medium	1.21 (1.12,1.33) ^*^	2.81 (2.49,3.18) ^**^	1.25 (1.15,1.36) ^**^	3.15 (2.78,3.57) ^**^	1.35 (1.23,1.47) ^**^	3.07 (2.71,3.48) ^**^
Chronotype						
Intermediate chronotype	2.71 (2.40,3.06) ^**^	5.21 (4.56,5.95) ^**^	2.82 (2.49,3.19) ^**^	5.73 (5.01,6.56) ^**^	2.71 (2.39,3.08) ^**^	5.24 (4.57,6.01) ^**^
Eveningness	1.52 (1.38,1.66) ^**^	1.75 (1.57,1.96) ^**^	1.57 (1.43,1.73) ^**^	1.90 (1.70,2.12) ^**^	1.57 (1.43,1.72) ^**^	1.83 (1.64,2.05) ^**^

Model 1: uncontrolled covariate; Model 2: controlled gender and age; Model 3: Control gender, age, parents' education level, family residence, only child, family economic status, number of friends and academic performance; *P < 0.05; **P < 0.01

As shown in Supplement Table 4 (pages 10–11), higher levels of SERF, intermediate chronotype, and eveningness were associated with higher cooccurrence of HRBs (total SERF OR = 7.36, 95% CI: 4.50, 12.05; eveningness OR = 12.34, 95% CI: 5.91, 25.77; and intermediate chronotype OR = 4.36, 95% CI: 3.08, 6.15). After adjusting for covariates, these results were identical (all *p*-values < 0.01).

As shown in Supplement Table 5 (page 12), we also explored the interactions between chronotype, SERF, and clustering of HRBs and mental health. Higher SERF and eveningness were associated with higher suicide and NSSI scores (OR = 27.84, 95% CI: 22.03, 35.19) and a greater number of mental health issues (OR = 18.46, 95% CI: 13.16, 25.88).

### Moderate mediation analysis

We performed moderated mediation analyses by controlling the covariates noted previously. The results demonstrated that the effect of SERFs on the clustering of HRBs was significantly moderated by chronotype. In particular, the mediation of mental health on HRBs was significantly moderated by chronotype (Supplement Table 6, page 12). Furthermore, morningness predicted more severe mental health issues. However, while morningness enhanced the effect of mental health, it weakened the effect of SERFs (Supplement Table 7 and Supplement Fig. 1, page 13).

We also performed moderated mediation analyses by controlling the covariates noted previously for HRBs cooccurrence. The results demonstrated that the effect of SERFs on mental health was significantly moderated by chronotype (SERF*chronotype: β = 0.071, *p* < 0.01). Specifically, the mediation of mental health on HRBs was significantly moderated by chronotype (mental health*chronotype: β = ˗0.001, *p* < 0.01; Supplement Table 8, page 13). Similar results were shown in supplement Table 9 and supplement Fig. 2 (pages 14–15).

## Discussion

### Principal findings

This study examined the correlation between chronotypes and SERFs and their respective associations with mental health and HRBs in a school-based sample of adolescents. This study used LCA to deduce adolescent HRB patterns from pre-specified behavioral variables. Multiple indicators of HRBs were examined using the LCA method within a theoretically derived model of SES influence on class membership. In our study, the prevalence of clustering of HRBs was 21.3%. Our study also explored the relationship in different dimensions of SERFs, including spatial and chronosystem. Moreover, an interaction between chronotypes and SERFs and HRBs and mental health was observed in this study. We examined the mediating effect of mental health and the moderating role of the chronotype on the relationship between HRBs and SERFs. Our results strongly supported the hypotheses regarding different factors and potential mechanisms associated with adolescent HRBs.

### The correlation between SERFs and psychological and behavioral problems

SERFs refer to risk factors and events that are not conducive to individual development and adaptation when an individual's living environment includes a variety of backgrounds, and these factors and events may increase the risk of negative outcomes [30]. Although these frameworks may vary, most of them reflect the principles of the social ecological model, a simple but far-reaching framework for public health [31, 32]. Based on the research on SERFs, some studies have reported that the high social-ecological risk group of adolescents is at higher risk of psychological, emotional, and behavioral problems [23, 33]. Individuals are often faced with multiple risks at the same time, if only focus on a single area is inconsistent with the reality of life. In other words, studies on the risk factors that lead to adolescent health risk behaviors are mostly explored from a single or minority level. However, adolescents will be affected by multiple risks such as family, community, school and peers during their growth, and their psychological problems are rarely caused by a single risk factor. Sometimes different risk factors do not act independently. More often, multiple risk factors at the same time [33, 34]. Our results demonstrated that cumulative SERFs were positively correlated with clustering of HRBs and HRB cooccurrence, consistent with ecological system theory. The concept of cumulative risk has also gained support across social and cognitive domains following the pioneering research on ecological risk [13]. Cumulative risk models suggest that it is better to predict individual outcomes by exploring the accumulation of risk factors than by focusing on adverse outcomes of a single indicator.

### Complex associations between SERFs, chronotype, mental health and HRBs

Our study further exaimed the association between chronotypes, SERFs, mental health and HRBs. First, eveningness was found to be associated with higher HRBs and higher rates of mental health issues, which is consistent with previous findings in college students [35] and applies these to adolescents in this study. Second, previous studies found that certain social factors (such as parental or peer behavior) and individual factors (such as low self-esteem) were also associated with higher HRBs [36]; in addition, Williamson et al. found cumulative socio-ecological risk indexes were most associated with increased middle childhood sleep problems, this study systematically evaluated the interaction of socio-ecological risks, sleep problems, and individual adolescent factors, as well as the direct and indirect effects of maternal and paternal risk factors [37]. We performed a latent class analysis of HRBs based on the previous literature [25]. Our analysis was informed by hypotheses derived from the social development model between SERFs and HRBs [38]. Moreover, our results further provided a theoretical basis for the further study of moderated mediation analyses and their role in the significance of mental health, SERFs, chronotypes and HRBs. Third, there was an interaction between higher-risk SERFs, eveningness and higher HRBs, negative mental health symptoms. This means that adolescents who experience high-risk psychosocial environments where life and rest are not in harmony, such as circadian rhythm disturbances, may have a higher incidence of physical and mental health problems [14]. These results are similar to those of previous studies, the study of social ecological psychology aims to explore how specific features of social ecology lead to a state of mind, which can also affect people's emotions or behaviors [39]. Possible mechanisms underlying this have been explained by the diathesis-stress model [40] or comprehensive social-ecological diathesis-stress model [41].

The interaction and correlation results also suggested that a positive social environment can counteract the physical and mental health effects caused by an individual’s (potentially genetically) disturbed circadian rhythm [42]. Our study addressed this question from the perspective of chronotype, a trait related to circadian rhythms [43]. Wills et al. developed the moderator model to explore the relationship between chronotype score (an independent variable), depression (outcome), and social support (the moderator and mediator) and found that chronotype was associated with depression in individuals with low social support from friends and teammates [44]. This result further verifies that there is an association between SERFs, chronotype and HRBs. We also explored the moderating effects of chronotype by dividing the relationship between mental health, chronotype and HRBs in three aspects from a social ecological system perspective. In our study, we found that high SERF scores were associated with higher HRBs and mental health issues. Possible reasons for this may be that evening chronotypes may be predisposed to mental health disturbances, potentially through a behavioral inhibition pathway [39]. Similarly, adolescents who experienced higher SERFs combined with eveningess, experienced more negative mental health effects and higher levels of HRBs. Eveningness may influence the HRB problems caused by SERFs. Moreover, eveningness may be a part of a broader temperamental [45] and personality [46] profile of adolescents that may predispose to mental health problems. Future research should focus on consideration of individual differences in temperament or personality.

Based on the mediate moderation analysis, it has been found that an individual's chronotype can interfere with the timing of working hours, school, or social schedules. One study discussed the association between adverse social events, such as adverse childhood experiences, chronotypes, and HRBs, such as smoking and alcohol consumption [47]. Potential SERFs and the resulting HRBs that affect mental health may be blocked or inhibited by morning type effects, that is, reasonable sleep habits can offset the adverse psychological outcomes brought by multi-level environment and unfavorable HRBs. These results confirm our previous findings exploring the mediating role of chronotype in mental health and the moderating role of chronotype in our use of multiple social contexts to measure the actual social reality of adolescents and their circadian habits (such as eveningness). This study also provided some new perspectives other than just life events to further investigate the association between circadian rhythm and SERFs, HRBs and mental health. A previous study indicated that the relationship between chronotype and experiences of negative life events may be mediated either by personality traits (neuroticism, risk-taking behavior, and externalizing behavior) or poor sleep quality [45, 48]. This concept offered a theoretical basis for our research, Through social ecological models, the description of mental and behavioral health can be understood from the perspective of holism, in which the individual is understood to be connected to historical and cultural backgrounds, family, community, land and even future generations in a highly relevant context [49]. Not only SERFs have an important effect on adolescent HRBs, but this association may be mediated by mental health, mediated by chronotype. Therefore, according to the results of this study, we should advocate ameliorating adolescents' living habits in the evening as much as possible, and provide them with more support at home, at school or in the community.

### Strengths and limitations

The strengths of this study included the followings: this was multicenter and multilevel design and the large number of study participants. Based on the previous concept of chronosystem, this study innovatively proposed factors that may affect adolescent HRBs, supplemented the original concept of ecological theory, and provided theoretical and practical basis for the subsequent expansion of the application of chronosystem.

However, some limitations should be noted. Because of the cross-sectional design, a casual association between SERFs and HRBs could not be established. Future longitudinal studies are needed to elucidate the association between the variables. Fifteen types of adolescent HRBs were examined in this study, although these did not include addiction, intentional injury, and unsafe behaviors, which are variables that have been included in other studies. However, we will consider these behaviors in a future study involving college students. All of the data used in this study were obtained from self-reported questionnaires; therefore, there were issues with subjectivity, validity, and reliability of the answers. This study only analyzed results from three cities; therefore, it is not clear how representative this sample is of the overall population in China. A follow-up survey will be conducted on population samples from different regions and cultures across China. In this study, the cumulative risk index was adopted to explore SERFs, which limited the universality of this study. Although this study focused on the association between SERFs and HRBs in certain fields, the possible application of this correlation in other fields still needs further investigation. Future research should explore the association between SERFs and HRBs in different fields.

## Conclusions

In our study, we found that adolescents who had high cumulative SERFs also experienced high-risk HRBs, and eveningess adolescents also had high-risk HRBs. An interactive positive association between cumulative SERFs, eveningness on HRBs, and mental health was also identified. In conclusion, the mental health of adolescents should be considered in mediate moderator analysis, and adolescents should be encouraged to improve their chronotype habits. This study focuses on the diversity of adolescents' living environment to provide a solid foundation for promoting the healthy growth of adolescents.

## Supplementary Information

**Additional file 1: Supplement table 1.** Items. **Supplement table 2.** Screening results of latent categories clustering of health risk behaviors. **Supplement table 3.** The prevalence characteristics and social ecological risk factosr and chronotype of mental health. **Supplement table 4.** The Logistic regression analysis between social ecological risks and co-occurrence of health risk behaviors in adolescents. **Supplement table 5.** The interaction between chronotype, SES, mental health and HRBs. **Supplement table 6.** Model characteristics for the conditional process analysis.** Supplement table 7.** Bootstrapped conditional direct and indirect effects. **Supplement Fig. 1.** Moderation mediate model (clustering of HRB). **Supplement table 8.** Model characteristics for the conditional process analysis. **Supplement table 9.** Bootstrapped conditional direct and indirect effects. **Supplement Fig. 2.** Moderation mediate model (HRB co-occurrence).

## Data Availability

The datasets generated and/or analysed during the current study are not publicly available but are available from the corresponding author on reasonable request.
