# Supplementary material for: Relationship between chronotype and mental behavioural health among adolescents: a cross-sectional study based on the social ecological system
Source: BMC Psychiatry. 2023 Jun 6;23:404. doi: 10.1186/s12888-023-04879-6 (PMC10243001; doi:10.1186/s12888-023-04879-6)
Supplement: Supplementary file 1 — Additional file 1: Supplement table 1. Items. Supplement table 2. Screening results of latent categories clustering of health risk behaviors. Supplement table 3. The prevalence characteristics and social ecological risk factosr and chronotype of mental health. Supplement table 4. The Logistic regression analysis between social ecological risks and co-occurrence of health risk behaviors in adolescents. Supplement table 5. The interaction between chronotype, SES, mental health and HRBs. Supplement table 6. Model characteristics for the conditional process analysis. Supplement table 7. Bootstrapped conditional direct and indirect effects. Supplement Fig. 1. Moderation mediate model (clustering of HRB). Supplement table 8. Model characteristics for the conditional process analysis. Supplement table 9. Bootstrapped conditional direct and indirect effects. Supplement Fig. 2. Moderation mediate model (HRB co-occurrence). [file 12888_2023_4879_MOESM1_ESM.doc]

**2 Methods**

**Settings**

Cross-sectional study design was used to conduct nationwide sample survey in China. They were mostly from grade 7-12. The first step is to sample the area. Based on China's geographical distribution, economic development level, and cooperation agreements with this research group, these cities can represent the characteristics of social, economic and cultural development in different regions of China, Beijing, Zhengzhou in Henan Province and Yangjiang in Guangdong province. The second step and third step are school sampling and class choosing. Two rural junior high schools and two urban junior high schools were randomly selected from each region. No less than 200 students were selected from each grade of each school for questionnaire survey, and no less than 4800 people were selected from each region. In each school, all students of 3 classes in each grade were selected for questionnaire survey.

**Sample size estimation**

Based on previous research experience, more than 3 of 18% of the 9 HRBs are used for reference, with the relative precision of 15% (*ɛ*), α=0.05, Z_1-α/2_ = 1.96. Using the following formula [1], the minimum sample size was 397 people. Consider a multicenter design, with a provincial, grade, rural-urban, etc. analysis and future follow-up needs. This minimum requirement was used for grade sampling in all provinces to ensure that the analysis was performed at multiple stratification levels by province. Therefore, a total of 15 600 people were surveyed in each school, with 400 participants from each school and half gender.


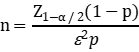


***Health risk behaviors***

1) Physical inactivity, the use of "Physical Activity rating Scale" (PARS-3) questionnaire including 3 items, the first is about the intensity of exercise, "in the past month, when you do physical exercise, most of the following intensity of exercise will be selected? The answers include: ① light exercise (such as walking on the playground, doing radio exercises, etc.); ② the movement of small intensity, low intensity (recreational recreational ball games, including the volleyball and table tennis, jogging, tai chi, etc.), ③ of moderate intensity, more intense lasting movement (such as cycling and running more than half an hour), ④ shortness of breath, sweating a lot of great strength, but not lasting movement properties of ball games (game), ⑤ rapid breathing, sweating a lot, high intensity of sustained and lasting exercise (such as running, spinning, swimming, etc.);

The second one is about the evaluation of the duration of exercise, "how many minutes does the activity of the above intensity last?" the answers include ① ≤ 10 minutes, ② 11-20 minutes, ③ 21-30 minutes, ④ 31-59 minutes, ⑤ ≥ 60 minutes;

The third is the frequency of physical activity, "how many times have you done these activities?" including ①1 month ≤ 1 time; ② 2 ~ 3 times a month; ③ 1~2 times a week; ④ three to five times a week; ⑤ About once a day;

Calculate the physical activities of youth, the intensity and frequency of option value assignment for 1 ~ 5 minutes, the time assignment of 0 ~ 4 points, the calculating formula for physical activity the strength×time×frequency, in accordance with the 50 percentile is divided into low activity and high activity, P_50_ or less, for lack of physical activity > P_50_ for sufficient physical activity;

2) Vegetables and fruits (V&F): "How many servings of vegetables have you usually eaten per day in the last month?" And" How many servings of fruit have you usually eaten per day in the last month?" The answers were "no", "less than 1 portion", "1 portion", "2 portions", "3 portions or more", and "1 portion" and below were defined as insufficient intake of vegetables and fruits, and "2 portions" and above were defined as adequate intake of vegetables and fruits.

3) Smoking. "How many days in the past month have you smoked at least one cigarette?" The answers included "no", "1 or 2 days", "3 ~ 5 days", "6 ~ 9 days", "10 ~ 19 days", "20 ~ 29 days" and "all 30 days". "no" was defined as no smoking behavior, and "1 day" and more was defined as have smoking behavior.

4) Alcohol consumption: "How many days in the last month have you had at least one glass of wine (a glass of wine is equivalent to half a bottle/can of beer, a small glass of white wine, a glass of wine or yellow rice wine, rather than a sip)"; It also asked "If you're a girl, how many days do you have at least four drinks in an hour or two, and if you're a guy, how many days do you have at least five drinks in an hour or two?" The answers included "no", "1 day", "2 days", "3 ~ 5 days", "6 ~ 9 days", "10 ~ 19 days" and "more than 20 days". "no" was defined as no drinking behavior, and "1 day" and more than 20 days were defined as have drinking behavior.

5) Screen time: "In a typical day, how much time do you spend sitting, reclining, or lying down? (This includes time spent sitting studying, reading, watching TV, using the computer, resting, all static activities, but not sleeping)". weekday ST and weekend ST include "almost none", "less than 1 hour a day", "less than 2 hours a day", "less than 3 hours a day", "less than 4 hours a day" and "more than 4 hours". The no ST on weekday is defined as the no ST, and have ST is defined as the time with screen. Less than 1 hour a day on rest days was defined as short ST, ≥ 1 hour ST was defined as long ST.

6) Skipping breakfast: "How many days in the last 7 days have you eaten breakfast?" Less than 7 days was defined as insufficient breakfast intake, and 7 days was defined as adequate breakfast intake.

7) High consumption of fast food/takeaway: "How many times have you eaten fast food/takeaway (such as Ele. me, Humming bird, Meituan Takeaway, Baidu Takeaway and other fast food) in the last week"; the answers include "have not eaten", "1 ~ 2 times", "3 ~ 4 times" and "5 times or more". "Have not eaten" is defined as no takeaway/fast food consumption, and "have eaten once" or more is defined as takeaway/fast food consumption.

8) Sugar sweetened beverages (SSBs): "In the last month, you usually drank more than 250 ml (a can) of soft drinks (such as Coke, Sprite, Fanta, etc.) several times a week"; "No" was defined as SSB consumption, and "once" or more was defined as SSB consumption.

9) Suicidal ideation, suicidal plan and suicidal attempt: "Have you seriously considered suicide in the last year?" "Have you made a plan to commit suicide in the past year?" And" Have you committed suicide in the past year?" The answers included "no", "1 time", "2 ~ 3 times" and "4 or more times". No suicidal ideation, suicide planning and suicide attempt were all defined as "no", and suicide ideation, suicide plan and suicide attempt were all defined as "1 time" or more.

10) Non-suicidal self-injury (NSSI) : "In the last 12 months, have you ever intentionally hurt yourself without the intention of killing yourself?" The article listed several methods of NSSI: hitting yourself with the fist or palm, pulling your hair, hitting hard objects with your head or fist, pinching or scratching yourself, biting yourself, cutting yourself or stabbing yourself including 12 questions. For those who have hurt themselves, ask them how often they hurt themselves. The frequency of each NSSI is the total number of NSSI [2]. "no" is defined as no NSSI, and "1" or more is defined as having NSSI.

*Social ecological risk factors*

The social ecological risk factors (SERFs) has 50 items, divided into seven dimensions, including individual, family, school, community, policy, culture and chronosystem profiles. , response scores ranged from 1= “absolutely agree”, 2 = “generally agree”, 3 = “somewhat agree”, 4 = “generally disagree”, and 5 = “absolutely disagree”. Analysis of each dimension and the total score of SERFs, the calculation of each dimension score first, according to the score below the 25th percentile and more than 75 percentile, and between the two, set the score of each dimension to three classification variables, respectively named as low risk, medium risk and high risk, including ≤ P_25_ indicate less low risk, > P_25_ ~ ≤ P_75_ indicate medium risk, and > P_75_ indicate high risk [Evans et al., 2013]. When calculating the total score, add the total score of each dimension and divide it into low risk, medium risk and high risk according to the above method. The Cronbach’s α coefficient for the SERF was 0.898 in the present study. Detailed items were shown in **sTable 1**.

| **Supplement table 1**  **Items** |
| --- |
| 1. I can always solve problems if I try my best |
| 2. I can stick to some things very well, such as physical exercise and extracurricular reading |
| 1. I am confident that I can deal effectively with anything that comes my way |
| 4. I feel like I can't accomplish a lot of things |
| 5. You like to keep something you're not happy about inside, but you can't forget it |
| 6. I can self-soothe when I encounter setbacks |
| 7. When a painful event occurs, use positive actions to alleviate the pain |
| 8. You often lose your temper by blaming others for unhappy things |
| 9. I have a good heart to heart with my parents |
| 10. My parents respect my feelings |
| 11. My parents know when I'm upset about something |
| 12. Many of my troubles are unknown to my parents |
| 13. My family members get along well with each other |
| 14. My parents are always quarrelling about me |
| 15. My parents support my healthy behaviors |
| 16. My parents often scold me for every little thing |
| 17. I feel very uncomfortable at school |
| 18. There are many things I like in school |
| 19. I think I can fit in the school life well |
| 20. When some of my classmates bullied me when I was young, my classmates would stop me |
| 21. I think teachers' attitude towards me often depends on my academic performance |
| 22. The teacher's lecture was as dry as a whistle |
| 23 I think the teacher is fair to every student |
| 24 My teacher likes me very much |
| 25 My classmates and I often study together |
| 26 I don't think the standard of education in schools is good |
| 27. I was attacked by my classmates at school |
| 28. I get on well with my classmates |
| 29. The air in our living places (including dormitories) often smells bad |
| 30. Living place (including dormitory) is close to the road, so the noise affects my rest |
| 31. There are no special places for people to exercise around their living areas (including dormitories) |
| 32. Strangers often behave in dangerous ways around their living quarters (including dormitories) |
| 33. Accommodation (including dormitories) is not co |
| 34. The school has a reward policy for students who take part in the sports meeting |
| 35. Schools have penalties for intentional damage to public property |
| 36. School meals are more hygienic |
| 37. The school has strict supervision over Internet cafes and entertainment venues around it |
| 38. The community has strict supervision over Internet cafes and entertainment venues around them |
| 39. The community encourages participation in community activities |
| 40. Parents divorced in recent 3 years |
| 41. Family members convicted of criminal or other offences in the last three years |
| 42. In the past three years, parents have adopted a younger brother or sister |
| 43. The family has moved to a new place to live or go to school in the last 3 years |
| 44. The family has been suffering from serious illness for the last three years |
| 45. I think the things my idol does (tattoos, baring midriff) are acceptable to me |
| 46. I think going out is better than playing live streaming or Douyin at home |
| 47. I'll buy anything expensive if I like it |
| 48. I can have a face-lift |
| 49. I think chatting on mobile phones is more fun than chatting in person, for example, we can send memes |
| 50. I think premarital sex is ok as long as two people are mutually agreeable |

**Sensitivity analysis**

In this study, sensitivity analysis was used to test the robustness of the model: (1) model 1 did not control for covariables; model 2 controlled for gender and age [3]; model 3 controlled for gender, age, parental education level, residential areas, only child, family economic status, number of friends and academic record; (2) Logistic regression was performed for each dimension of SERF with clustering of HRBs; (3) Explore the correlation between various dimensions of SERF and co-occurrence of HRB, and calculate the co-occurrence of HRBs using the following methods: physical inactivity, smoking, drinking, weekday and weekend screen time, takeaway, fast food, SSB, skip breakfast, vegetables, fruits, the NSSI and suicide ideation, suicide plan and suicide attempt of 15 kinds of HRBs carried out in accordance with the presence of risk classification, divided into two, according to standards according to the variable will occur for each HRB danger, the number of all HRBs in each research object was added up to form a "co-occurrence index" [4], which divided the co-occurrence of HRBs into 0, 1-3 and 4 or more types.

**Reference**

1 OpenEpi Toolkit Shell for Developing New Applications. Available from: http://www.openepi.com/SampleSize/SSPropor.htm.

2 Wan Y, Chen R, Ma S, et al. Associations of adverse childhood experiences and social support with self-injurious behaviour and suicidality in adolescents. Br J Psychiatry. 2019;214(3):146-152. doi:10.1192/bjp.2018.263.

3 Yamaguchi N, Mahbub MH, Takahashi H, et al. Plasma free amino acid profiles evaluate risk of metabolic syndrome, diabetes, dyslipidemia, and hypertension in a large Asian population. Environ Health Prev Med. 2017;22(1):35. doi:10.1186/s12199-017-0642-7.

4 Hausdorf K, Eakin E, Whiteman D, et al. Prevalence and correlates of multiple cancer risk behaviors in an Australian population-based survey: results from the Queensland Cancer Risk Study. Cancer Causes Control, 2008, 19(10):1339-1347.

**3 Results**

| Supplement table 2 Screening results of latent categories clustering of health risk behaviors | | | | | | |
| --- | --- | --- | --- | --- | --- | --- |
|  | AIC | BIC | aBIC | LMR-LRT | BLRT | Entropy |
| 1 |  |  |  | ＜0.001 | ＜0.001 |  |
| 2 | 241444.85 | 241684.55 | 241586.64 | ＜0.001 | ＜0.001 | 0.854 |
| 3 | 236941.73 | 237305.15 | 237555.78 | ＜0.001 | ＜0.001 | 0.746 |
| 4 | 235292.19 | 235779.33 | 235579.12 | ＜0.001 | ＜0.001 | 0.749 |
| 5 | 234213.31 | 234824.16 | 234573.11 | ＜0.001 | ＜0.001 | 0.701 |
| 6 | 233347.19 | 234081.76 | 233779.86 | ＜0.001 | ＜0.001 | 0.709 |
| 7 | 2327.65.59 | 233623.87 | 233271.12 | ＜0.001 | ＜0.001 | 0.733 |
| 8 | 232070.70 | 233052.70 | 232649.11 | ＜0.001 | ＜0.001 | 0.693 |
| 9 | 231696.46 | 232802.18 | 232347.73 | ＜0.001 | ＜0.001 | 0.717 |

| Supplement table 3 The prevalence characteristics and social ecological risk factosr and chronotype of mental health | | | | |
| --- | --- | --- | --- | --- |
|  | Total | Low | High | *χ*^2^ value |
| Age |  | 15.22±1.75 | 15.05±1.63 | 3.77^**^ |
| Gender |  |  |  | 26.41^**^ |
| Male | 8390(49.8) | 7671(91.4) | 719(8.6) |  |
| Female | 8463(50.2) | 7539(89.1) | 924(10.9) |  |
| Residential area |  |  |  | 5.43 |
| Country | 2593(15.4) | 2336(90.1) | 257(9.9) |  |
| Town | 3212(19.1) | 2934(91.3) | 278(8.7) |  |
| Urban | 11048(65.6) | 9940(90.0) | 1108(10.0) |  |
| Only child |  |  |  | 7.03^**^ |
| Yes | 5710(33.9) | 5105(89.4) | 605(10.6) |  |
| No | 11143(66.1) | 10105(90.7) | 1038(9.3) |  |
| Father's education |  |  |  | 68.68^**^ |
| No father | 189(1.1) | 139(73.5) | 50(26.5) |  |
| Below primary school level | 505(3.0) | 452(89.5) | 53(10.5) |  |
| Primary school | 1325(7.9) | 1171(88.4) | 154(11.6) |  |
| Junior high school | 6022(35.7) | 5454(90.6) | 568(9.4) |  |
| Senior high school | 4902(29.1) | 4454(90.9) | 448(9.1) |  |
| College school | 3910(23.20 | 3540(90.5) | 370(9.5) |  |
| Mother's education |  |  |  | 63.25^**^ |
| No mother | 115(0.7) | 80(69.6) | 35(30.4) |  |
| Below primary school level | 744(4.4) | 656(88.2) | 88(11.8) |  |
| Primary school | 1679(10.0) | 1511(90.0) | 168(10.0) |  |
| Junior high school | 6036(35.8) | 5477(90.7) | 559(9.3) |  |
| Senior high school | 4634(27.5) | 4173(90.1) | 461(9.9) |  |
| College school | 3645(21.6) | 3313(90.9) | 332(9.1) |  |
| Family economic status |  |  |  | 152.42^**^ |
| Very bad | 475(2.8) | 366(77.1) | 109(22.9) |  |
| Worse | 1644(9.8) | 1422(86.5) | 222(13.5) |  |
| Medium | 11677(69.3) | 10672(91.4) | 1005(8.6) |  |
| Better | 2431(14.4) | 2212(91.0) | 219(9.0) |  |
| Very good | 626(3.7) | 538(85.9) | 88(14.1) |  |
| Friends number |  |  |  | 513.95^**^ |
| No | 553(3.3) | 376(68.0) | 177(32.0) |  |
| 1-2 | 4096(24.3) | 3501(85.5) | 595(14.5) |  |
| 3-5 | 6949(41.2) | 6432(92.6) | 517(7.4) |  |
| 6 or more | 5255(31.2) | 4901(93.3) | 354(6.7) |  |
| Academic record |  |  |  | 180.22^**^ |
| Bad | 4087(24.3) | 3467(84.8) | 620(15.2) |  |
| Medium | 10316(61.2) | 9488(92.0) | 828(8.0) |  |
| Good | 2450(14.5) | 2255(92.0) | 195(8.0) |  |
| Individual |  |  |  | 941.33^**^ |
| high | 4883(29.0) | 3895(79.8) | 988(20.2) |  |
| medium | 5610(33.3) | 5156(91.9) | 454(8.1) |  |
| low | 6360(37.7) | 6159(96.8) | 201(3.2) |  |
| Family |  |  |  | 734.91** |
| high | 4644(27.6) | 3747(80.7) | 897(19.3) |  |
| medium | 6380(37.9) | 5855(91.8) | 525(8.2) |  |
| low | 5829(34.6) | 5608(96.2) | 221(3.8) |  |
| School |  |  |  | 741.77** |
| high | 5251(31.2) | 4279(81.5) | 972(18.50 |  |
| medium | 5732(34.0) | 5261(91.8) | 471(8.2) |  |
| low | 5870(34.8) | 5670(96.6) | 200(3.4) |  |
| Community |  |  |  | 320.19** |
| high | 4520(26.8) | 3779(83.6) | 741(16.4) |  |
| medium | 6335(37.6) | 5819(91.9) | 516(8.10 |  |
| low | 5998(35.6) | 5612(93.6) | 386(6.4) |  |
| Policy |  |  |  | 120.21** |
| high | 4597(27.3) | 3961(86.2) | 636(13.8) |  |
| medium | 6320(37.5) | 5809(91.9) | 511(8.1) |  |
| low | 5936(35.2) | 5440(91.6) | 496(8.4) |  |
| Culture |  |  |  | 396.07** |
| high | 4860(28.8) | 4049(83.3) | 811(16.7) |  |
| medium | 6186(36.7) | 5680(91.8) | 506(8.2) |  |
| low | 5807(34.5) | 5481(94.4) | 326(5.6) |  |
| Chronosystem |  |  |  | 1176.36** |
| high | 5456(32.4) | 4319(79.2) | 1137(20.8) |  |
| medium | 5919(35.1) | 5546(93.7) | 373(6.3) |  |
| low | 5478(32.5) | 5345(97.6) | 133(2.4) |  |
| Total score |  |  |  | 275.79** |
| high | 2978(17.7) | 2448(82.2) | 530(17.8) |  |
| medium | 5209(30.9) | 4739(91.0) | 470(9.0) |  |
| low | 8666(51.4) | 8023(92.6) | 643(7.4) |  |
| Chronotype |  |  |  | 365.29** |
| Eveningness | 2767(16.4) | 2225(80.4) | 542(19.6) |  |
| Intermediate | 10482(62.2) | 9647(92.0) | 835(8.0) |  |
| Morningness | 3604(21.4) | 3338(92.6) | 266(7.4) |  |
| ^*^p <0.05, ^**^p < 0.01 | | | | |

| Supplement table 4 The Logistic regression analysis between social ecological risks and co-occurrence of health risk behaviors in adolescents | | | | | | | | |
| --- | --- | --- | --- | --- | --- | --- | --- | --- |
| Social ecological sys | Model 1 | |  | Model 2 | |  | Model 3 | |
|  | 1～3 | ≥ 4 |  | 1～3 | ≥ 4 |  | 1～3 | ≥4 |
| Individual |  |  |  |  |  |  |  |  |
| High | 1.64（1.003,2.69）^*^ | 4.85（2.97,7.92）^**^ |  | 1.59（0.97,2.61） | 4.70（2.87,7.69）^**^ |  | 1.61（0.96,2.69）^**^ | 4.68（2.80,7.83）^**^ |
| Medium | 1.52（1.02,2.24）^*^ | 2.49（1.68,3.67）^**^ |  | 1.47（0.99,2.17） | 2.39（1.62,3.53）^**^ |  | 1.48（0.99,2.21） | 2.40（1.61,3.58）^**^ |
| Family |  |  |  |  |  |  |  |  |
| High | 1.02（0.66,1.56） | 2.93（1.91,4.50）^**^ |  | 1.04（0.68,1.61） | 3.07（2.0,4.71）^**^ |  | 1.04（0.67,1.62） | 2.98（1.91,4.63）^**^ |
| Medium | 1.63（1.09,2.43）^*^ | 2.75（1.84,4.09）^**^ |  | 1.62（1.09,2.42）^*^ | 2.73（1.83,4.08）^**^ |  | 1.61（1.07,2.41）^*^ | 2.68（1.79,4.02）^**^ |
| School |  |  |  |  |  |  |  |  |
| High | 1.99（1.17,3.39）^*^ | 5.98（3.52,10.15）^**^ |  | 1.78（1.04,3.03）^*^ | 5.16（3.03,8.80）^**^ |  | 1.78（1.02,3.09）^*^ | 5.16（2.97,8.96）^**^ |
| Medium | 1.15（0.80,1.66） | 1.83（1.27,2.63）^**^ |  | 1.06（0.73,1.53） | 1.64（1.14,2.37）^**^ |  | 1.06（0.73,1.54） | 1.63（1.12,2.38）^*^ |
| Community |  |  |  |  |  |  |  |  |
| High | 1.26（0.82,1.93） | 2.41（1.57,3.70）^**^ |  | 1.13（0.73,1.75） | 2.26（1.51,3.38）^**^ |  | 1.12（0.72,1.75） | 2.22（1.47,3.33）^**^ |
| Medium | 1.79（1.20,2.68）^**^ | 2.53（1.70,3.78）^**^ |  | 1.64（1.10,2.46）^**^ | 2.11（1.37,3.25）^**^ |  | 1.62（1.08,2.44）^*^ | 2.07（1.33,3.21）^**^ |
| Policy |  |  |  |  |  |  |  |  |
| High | 3.22（1.79,5.79）^**^ | 7.09（3.95,12.72）^**^ |  | 2.85（1.57,5.16）^**^ | 6.01（3.33,10.86）^**^ |  | 2.92（1.61,5.32）^**^ | 6.01（3.31,10.94）^**^ |
| Medium | 1.94（1.33,2.84）^**^ | 2.69（1.85,3.93）^**^ |  | 1.78（1.21,2.60）^**^ | 2.39（1.63,3.49）^**^ |  | 1.78（1.21,2.63）^**^ | 2.38（1.62,3.50）^**^ |
| Culture |  |  |  |  |  |  |  |  |
| High | 2.01（1.17,2.43）^**^ | 8.06（4.72,13.75）^**^ |  | 1.70（0.98,2.94） | 6.87（3.97,11.88）^**^ |  | 1.72（0.99,2.98） | 6.94（4.01,12.02）^**^ |
| Medium | 1.86（1.26,2.77）^**^ | 3.81（2.57,5.66）^**^ |  | 1.64（1.10,2.46）^*^ | 3.37（2.25,5.04）^**^ |  | 1.64（1.09,2.46）^*^ | 3.35（2.24,5.02）^**^ |
| Chronosystem |  |  |  |  |  |  |  |  |
| High | 1.43（0.82,2.48） | 2.53（1.46,4.38）^**^ |  | 1.45（0.83,2.52） | 2.59（1.49,4.49）^**^ |  | 1.45（0.83,2.52） | 2.53（1.46,4.39）^**^ |
| Medium | 1.31（0.89,1.93） | 1.63（1.11,2.41）^*^ |  | 1.33（0.90,1.97） | 1.67（1.13,2.47）^*^ |  | 1.31（0.89,1.95） | 1.64（1.11,2.43）^*^ |
| Total score |  |  |  |  |  |  |  |  |
| High | 1.57（0.96,2.57） | 7.36（4.50,12.05）^**^ |  | 1.38（0.84,2.27） | 1.35（0.92,1.99） |  | 1.38（0.82,2.32） | 6.57（3.92,11.03）^**^ |
| Medium | 1.48（1.01,2.17）^*^ | 3.13（2.13,4.59）^**^ |  | 6.34（3.85,10.42）^**^ | 2.82（1.92,4.16）^**^ |  | 1.34（0.90,1.99） | 2.86（1.93,4.25）^**^ |
| Chronotype |  |  |  |  |  |  |  |  |
| Intermediate | 2.06（1.46,2.91）^**^ | 4.36（3.08,6.15）^**^ |  | 1.85（1.30,2.63）^**^ | 3.79（2.66,5.38）^**^ |  | 1.84（1.30,2.62）^**^ | 3.72（2.61,5.29）^**^ |
| Eveningness | 2.22（1.06,4.65）^*^ | 12.34（5.91,25.77）^**^ |  | 1.98（0.94,4.16） | 10.64（5.08,22.30） |  | 1.98（0.94,4.17） | 10.17（4.84,21.35）^**^ |

Model 1: uncontrolled covariate; Model 2: controlled gender and age; Model 3: Control gender, age, parents' education level, family residence, only child, family economic status, number of friends and academic performance；*，*P*＜0.05；**，*P*＜0.01.

| Supplement table 5 The interaction between chronotype, SES, mental health and HRBs | | | | | |
| --- | --- | --- | --- | --- | --- |
| Variables | HRBs clustering | |  | Mental health | |
|  | Medium | High |  | Low | High |
| SERF*Chronotype |  |  |  |  |  |
| High*eveningness | 3.72(2.10,4.48)^**^ | 27.84(22.03,35.19)^**^ |  | 1 | 18.46(13.16,25.88)^**^ |
| High*intermediate | 3.21(2.65,3.89)^**^ | 7.45(5.70,9.75)^**^ |  | 1 | 5.25(3.55,7.77)^**^ |
| High*morningness | 2.40(1.88,3.05)^**^ | 5.38(3.85,7.52)^**^ |  | 1 | 1.97(1.09,3.56)^*^ |
| Medium*eveningness | 2.26(1.95,2.61)^**^ | 12.55(10.13,15.55)^**^ |  | 1 | 9.09(6.52,12.69)^**^ |
| Medium*intermediate | 1.88(1.65,2.15)^**^ | 4.72(3.81,5.84)^**^ |  | 1 | 2.99(2.12,4.21)^**^ |
| Medium*morningness | 1.60(1.40,1.82)^**^ | 1.66(1.322.09)^**^ |  | 1 | 1.28(0.88,1.88)^**^ |
| Low*eveningness | 1.60(1.27,2.02)^**^ | 9.34(7.17,12.17)^**^ |  | 1 | 13.79(9.55,19.91)^**^ |
| Low*intermediate | 1.34(1.11,1.61)^**^ | 3.52(2.72,4.55)^**^ |  | 1 | 3.45(2.325.13)^**^ |
| Low*morningness | 1 | 1 |  | 1 | 1 |
| The model was controlled for age, gender, grade, parental education, family economy, numbers of friends, residential areas, academic record. ^*^p <0.05, ^**^p < 0.01 | | | | | |

| Supplement table 6 Model characteristics for the conditional process analysis. | | | | | | |
| --- | --- | --- | --- | --- | --- | --- |
| Variables | Mental health (MH) | | | HRB clustering | | |
|  | B | t value | *P* value | B | t value | *P* value |
| SERF | 3.26 | 20.06 | **＜0.01** | 0.05 | -6.19 | **＜0.01** |
| Chronotype | 0.53 | -9.51 | **＜0.01** | -0.02 | -3.0 | **＜0.01** |
| SES*Chronotype | -0.071 | 6.80 | **＜0.01** | -0.0001 | 0.27 | ＞0.05 |
| MH |  |  |  | 0.013 | 11.19 | **＜0.01** |
| MH*Chronotype |  |  |  | -0.0002 | -2.39 | **＜0.05** |
| R^2^ | 0.21 | | | 0.19 | | |
| F | 379.29 | | | 277.14 | | |
| Mediate variables: HRB, moderated variables: MEQ, independent variables: SERF, dependent variables: mental health symptoms. The model was controlled for age, gender, grade, parental education, family economy, numbers of friends, residential areas, academic record. | | | | | | |

| Supplement table 7 Bootstrapped conditional direct and indirect effects. | | | | | |
| --- | --- | --- | --- | --- | --- |
|  |  | | HRB clustering | | |
| Direct effect |  |  | Effect | SE | (LL,UL) |
|  | Predictor | SERF |  |  |  |
|  | Moderator (Chronotype ) | Low | -0.0466 | 0.0027 | -0.052,-0.0413 |
|  |  | Medium | -0.0461 | 0.0020 | -0.0501,-0.0422 |
|  |  | High | -0.0457 | 0.0026 | -0.0508, -0.0405 |
| Indirect effect |  |  | Effect | SE | (LL,UL) |
|  | Predictor | HRB |  |  |  |
|  | Mediator (MH) | Low | 11.21 | -0.0274 | -0.0303, -0.0250 |
|  |  | Medium | 14.74 | -0.0231 | -0.0251, -0.0213 |
|  |  | High | 18.28 | -0.0191 | -0.0217,- 0.0170 |


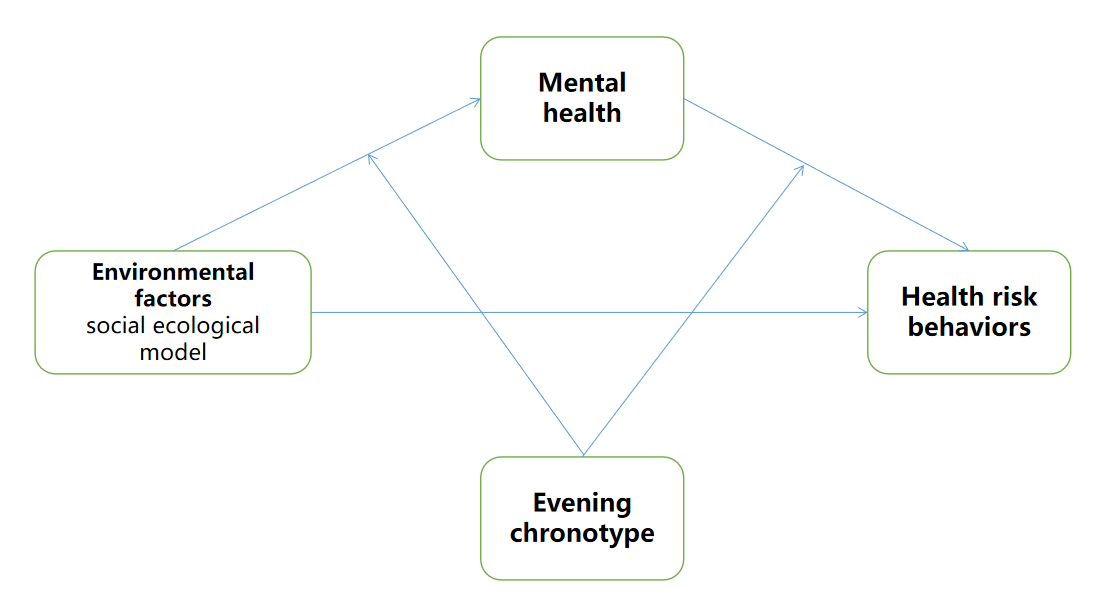


Supplement Fig. 1 Moderation mediate model (clustering of HRB)

| Supplement table 8 Model characteristics for the conditional process analysis. | | | | | | |
| --- | --- | --- | --- | --- | --- | --- |
| Variables | Mental health (MH) | | | HRB co-occurrence | | |
|  | B | t value | *P* value | B | t value | *P* value |
| SERF | 3.26 | 20.06 | **＜0.01** | 0.20 | 10.30 | **＜0.01** |
| Chronotype | 0.53 | 3.54 | **＜0.01** | -0.073 | -6.52 | **＜0.01** |
| SERF*Chronotype | 0.071 | 6.80 | **＜0.01** | -0.0026 | -2.05 | **＜0.05** |
| MH |  |  |  | 0.029 | 9.97 | **＜0.01** |
| MH*Chronotype |  |  |  | -0.001 | -2.98 | **＜0.01** |
| R^2^ | 0.21 | | | 0.25 | | |
| F | 379.29 | | | 399.23 | | |
| Mediate variables: HRB, moderated variables: MEQ, independent variables: SERF, dependent variables: mental health symptoms. The model was controlled for age, gender, grade, parental education, family economic status, numbers of friends, residential areas, academic record. | | | | | | |

| Supplement table 9 Bootstrapped conditional direct and indirect effects. | | | | | |
| --- | --- | --- | --- | --- | --- |
|  |  | | HRB co-occurrence | | |
| Direct effect |  |  | Effect | SE | (LL,UL) |
|  | Predictor | SERF |  |  |  |
|  | Moderator (Chronotype ) | Low | 0.170 | 0.0068 | 0.1567, 0.1833 |
|  |  | Medium | 0.1609 | 0.0050 | 0.1512, 0.1707 |
|  |  | High | 0.1519 | 0.0065 | 0.1391, 0.1647 |
| Indirect effect |  |  | Effect | SE | (LL,UL) |
|  | Predictor | HRB |  |  |  |
|  | Mediator (MH) | Low | 0.0558 | 0.0036 | 0.0489, 0.0630 |
|  |  | Medium | 0.0455 | 0.0023 | 0.0411, 0.0503 |
|  |  | High | 0.0362 | 0.0028 | 0.0308, 0.0424 |


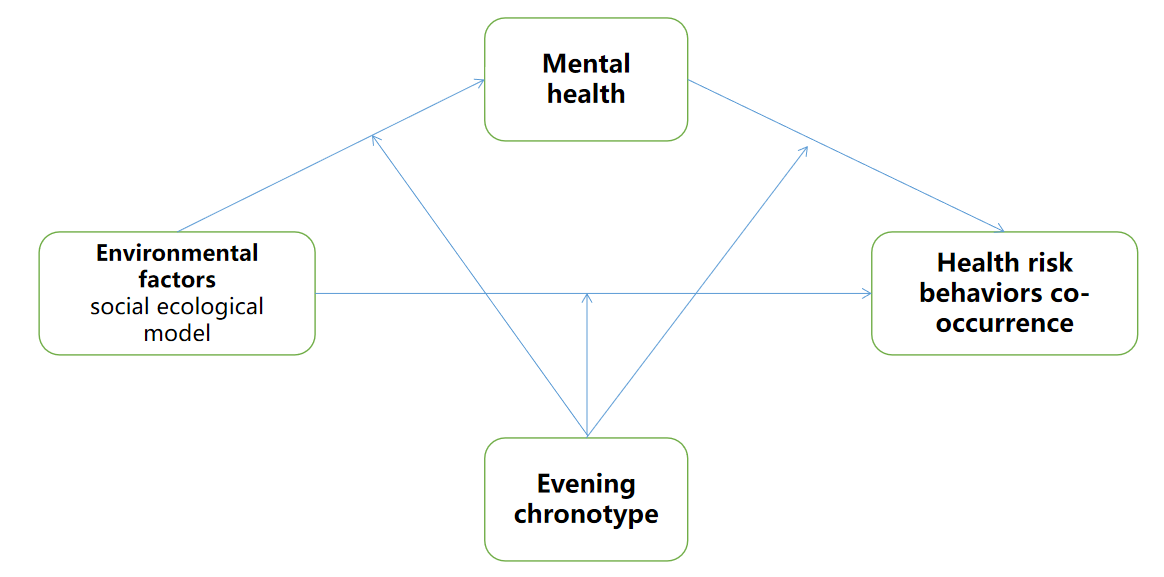


Supplement Fig. 2 Moderation mediate model (HRB co-occurrence)
